# Comprehensive analysis of multiprotein bridging factor 1 family genes and *SlMBF1c* negatively regulate the resistance to *Botrytis cinerea* in tomato

**DOI:** 10.1186/s12870-019-2029-y

**Published:** 2019-10-21

**Authors:** Xu Zhang, Zhixuan Xu, Lichen Chen, Zhonghai Ren

**Affiliations:** State Key Laboratory of Crop Biology, Shandong Collaborative Innovation Center of Fruit & Vegetable Quality and Efficient Production, Key Laboratory of Biology and Genetic Improvement of Horticultural Crops in Huang-Huai Region, Ministry of Agriculture, College of Horticultural Science and Engineering, Shandong Agricultural University, Tai’an, 271018 Shandong China

**Keywords:** Tomato, *MBF1*, Expression pattern, *SlMBF1c*, *Botrytis cinerea*

## Abstract

**Background:**

*Multiprotein bridging factor 1 s* (*MBF1s*) are members of the transcriptional co-activator family that have involved in plant growth, development and stress responses. However, little is known about the *Solanum lycopersicum MBF1* (*SlMBF1*) gene family.

**Results:**

In total, five *SlMBF1* genes were identified based on the tomato reference genome, and these genes were mapped to five chromosomes. All of the SlMBF1 proteins were highly conserved, with a typical MBF1 domain and helix-turn-helix_3 domain. In addition, the promoter regions of the *SlMBF1* genes have various stress and hormone responsive cis-regulatory elements. Encouragingly, the *SlMBF1* genes were expressed with different expression profiles in different tissues and responded to various stress and hormone treatments. The biological function of *SlMBF1c* was further identified through its overexpression in tomato, and the transgenic tomato lines showed increased susceptibility to *Botrytis cinerea* (*B. cinerea*). Additionally, the expression patterns of salicylic acid (SA)-, jasmonic acid (JA)- and ethylene (ET)- mediated defense related genes were altered in the transgenic plants.

**Conclusions:**

Our comprehensive analysis provides valuable information for clarifying the evolutionary relationship of the *SlMBF1* members and their expression patterns in different tissues and under different stresses. The overexpression of *SlMBF1c* decreased the resistance of tomato to *B. cinerea* through enhancing the gene expression of the SA-mediated signaling pathway and depressing JA/ET-mediated signaling pathways. These results will facilitate future functional studies of the transcriptional co-activator family.

## Background

Transcriptional regulation is a key step in the expression of genomic information during complex biological processes in all organisms. Transcriptional co-activators are important components of gene expression that function by interacting with transcription factors and/or other regulatory elements and the basal transcription machinery [1]. Multiprotein bridging factor 1 (MBF1) proteins are members of the transcriptional co-activator family and are highly conserved in eukaryotic organisms. MBF1 mediates the transcriptional activation of downstream genes by bridging regulatory transcription factors and TATA-box-Binding Protein [2]. MBF1 proteins are composed of a N-terminal domain, a conservative helix-turn-helix (HTH) domain and a short C-terminus [3]. The HTH domain is critical to maintain the functional activity of MBF1 [4].

Several *MBF1* genes have been identified in plants and have been shown to participate in plant growth, development and stress response. For example, *Arabidopsis thaliana* has three *MBF1* genes, and the expression levels of these genes have been found to be induced by various types of abiotic and biotic stress [2, 5, 6]. *Arabidopsis* plants that overexpress *Arabidopsis thaliana MBF1a* (*AtMBF1a*) show higher tolerance to salt stress and infection of pathogens, and they display a phenotype of hypersensitivity to Glucose [7]. The overexpression of *AtMBF1c* could enhance the tolerance to high temperature in *Arabidopsis* [8, 9]. The ectopic expression of *Vitis labrusca* x *V. vinifera MBF1* in *Arabidopsis* increased drought tolerance [10], and the ectopic expression the *Triticum aestivum MBF1c* improved thermotolerance in rice [11]. However, not all of the *MBF1* genes are positive regulators that can enhance tolerance to environmental stress in plants. For example, *Capsicum annuum MBF1* -overexpressing *Arabidopsis* lines have larger leaves but display sensitivity to cold and salt stress [12].

The tomato is one of the most widely cultivated vegetable crops in the world and a key model plant for the study of gene function [13]. However, the yield of tomato is seriously constrained by phytopathogens such as *Botrytis cinerea* (*B. cinerea*). Although the function of *SlER24*, a MBF1 family member, has been characterized and demonstrated to play an important role in tomato seed germination [14], the function of these genes except *SlER24* were few reported. In our study, in order to explore the gene number of the *SlMBF1* family in tomato, a systematic analysis was performed in tomato with the tomato genome database. A total of five SlMBF1 proteins were identified. The phylogenetic results and motif analysis showed that the SlMBF1 family was highly conserved. In addition, an analysis of promoter response elements and the expression profiling of the *SlMBF1s* revealed marked responses to various hormones and stresses. Moreover, we obtained transgenic lines in the tomato. The overexpression of *SlMBF1c* reduced the resistance of tomato to *B. cinerea*, suggesting *SlMBF1c* functions as a negative regulator in the tomato resistance to *B. cinerea*. Overall, the present study laid the foundation for the further study of *MBF1* genes, and their potentially use for trait improvement in the tomato.

## Results

### Identification and chromosomal location of *SlMBF1* genes in the tomato

To identify the putative *MBF1* genes in the tomato genome, we used the three *Arabidopsis* MBF1 protein sequences and the conserved MBF1 and HTH_3 domains as queries to search the tomato genome database using the BlastP program (Additional file 3: Table S3). A total of five putative SlMBF1 proteins were obtained with default parameters. Then, the existence of the conserved MBF1 and HTH_3 domains was confirmed by SMART and CD-Search. As described by Sanchez-Ballesta et al. [15], the four *SlMBF1* genes were named *SlMBF1a* to *c* and *SlER24*, and the newly identified *SlMBF1* gene was named *SlMBF1d*.

**Fig. 1:**
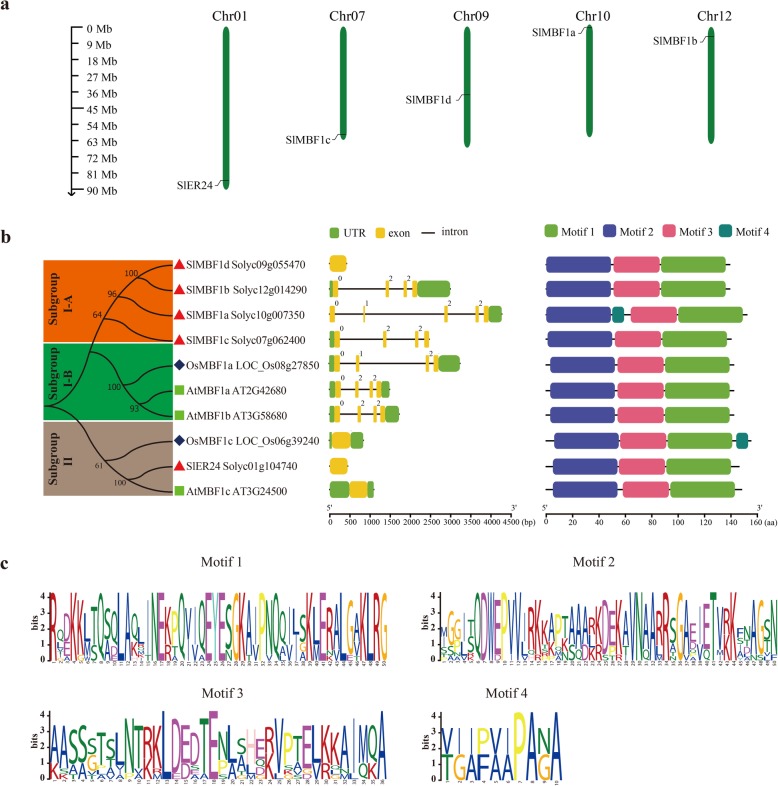
The analysis of the genomic locations, phylogenetic relationships, gene structures and conserved motifs. **a** Genomic locations of the five *SlMBF1* genes on five chromosomes. **b** The analysis of phylogenetic relationships, gene structures and conserved motifs in the *MBF1* genes from tomato, Arabidopsis and rice. The phylogenetic tree was constructed based on the full-length protein sequences of the five SlMBF1s, three AtMBF1 and two OsMBF1 proteins using MEGA 7.0 software. In the analysis of the gene structure, the number indicates the phases of corresponding introns. The UTR, exon, domain and motif are displayed in different colors, and the intron is displayed in a straight line. **c** The logos indicate the conserved motifs in the SlMBF1, AtMBF1 and OsMBF1 proteins

The molecular property analysis revealed that these SlMBF1 proteins display similar lengths (139 amino acid for SlMBF1a, SlMBF1b, SlMBF1d, and 146 amino acid for SlER24). The predicted molecular weights of the five SlMBF1 proteins ranged from 15.272 (SlMBF1b) to 16.033 (SlER24) Dalton (Da). The predicted pI values ranged from 9.95 (SlMBF1a and SlMBF1d) to 10.11 (SlER24). The gene IDs and genomic positions were summarized for these SlMBF1 proteins (Additional file 1: Table S1). By analyzing the genomic location information obtained from tomato genome database, these five *SlMBF1* genes were mapped on tomato chromosome 1, 7, 9, 10, 12, respectively (Fig. 1.a).

### Phylogenetic analysis, gene structure and conserved motifs of the *SlMBF1* genes

The full sequences of the five SlMBF1, three AtMBF1, and two OsMBF1 proteins were used to perform protein sequence alignment and phylogenetic analysis (Fig. 1b). These MBF1 proteins were defined as members of the other corresponding plants MBF1 subgroups [16]. Among these two subgroups, subgroup I is composed of four SlMBF1, one OsMBF1 and two AtMBF1 proteins, and subgroup II composed of one OsMBF1, one SlMBF1 and one AtMBF1 proteins. Due to evolutionary differences between these three species, subgroup I could be further divided into two groups, subgroup I-A and subgroup I-B. Among them, subgroup I-A included only four tomato SlMBF1 proteins and subgroup I-B included one OsMBF1 and two AtMBF1 proteins.

The gene structure analysis of the *MBF1* family genes from the tomato, *Arabidopsis* and rice were conducted and the results are consistent with the phylogenetic tree analysis. As shown in Fig. 1b, the number of exons in the *SlMBF1*, *AtMBF1* and *OsMBF1* genes ranges from one to five exons. We found that the two subgroups, subgroup II and subgroups I-B, have similar intron-exon structures (Fig. 1b). The three members, *OsMBF1c*, *SlER24* and *AtMBF1c*, in subgroup II contain one exon, and the members, *OsMBF1a*, *AtMBF1a* and *AtMBF1b*, in subgroups I-B four exons. However, in subgroups I-A, *SlMBF1b* and *SlMBF1c* contain four exons, while *SlMBF1a* five exons and *SlMBF1d* one exon (Fig. 1b).

The motif analysis of the MBF1 proteins was conducted and four distinct motifs were identified (Fig. 1b; c and Additional file 2: Table S2). Motif 2 and 3, which are MBF1 domains, and motif 1, which is an HTH_3 domain, were identified in all MBF1 proteins. Interestingly, motif 4 was only identified in the SlMBF1a and OsMBF1c proteins. Therefore, the similar motif distribution of the MBF1 proteins in these three model plants may promote to the prediction of the functions of MBF1s.

### Potential cis-elements in the promoters of *SlMBF1* genes

**Fig. 2:**
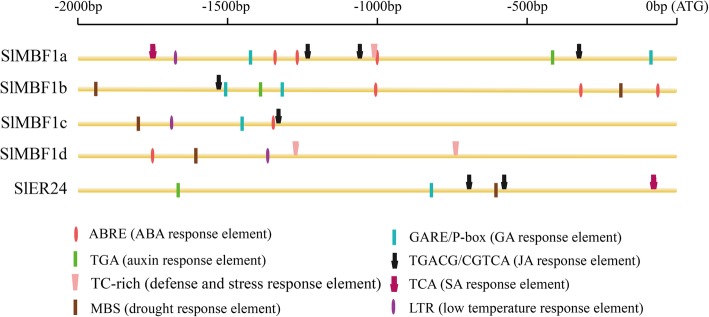
The promoter analysis of the *SlMBF1* members in the tomato. The potential cis-regulatory elements in the promoter regions 2.0-kb upstream of the *SlMBF1s* genes, particularly the elements related to stress responsiveness and plant hormones, are shown. Different shapes and colors indicate whether the motif exists in the plus or minus strand of the cis-acting elements

Previous studies have shown that many *MBF1* genes play regulatory roles in developmental processes and tolerance to environmental stresses in plants. To predict the putative functions of the SlMBF1 genes, the 2.0-kb promoter regions of the *SlMBF1* genes were isolated for the analysis of the potential cis-elements using the Plant-CARE database (Fig. 2), and many elements related to stress responsiveness and plant hormones were predicted. As shown in Fig. 2, the promoters of *SlMBF1* genes contain many stress elements: drought response element, low temperature response element and defense and stress response element. Moreover, hormone responsive elements including abscisic acid (ABA) response element, gibberellin (GA) response element, jasmonate acid (MeJA) response element, salicylic acid (SA) response element and auxin response element were also discovered in the *SlMBF1* promoters. These results suggest that the five *SlMBF1* genes may play important roles in the response to several hormones and various stresses.

### Expression pattern of the *SlMBF1* genes in different tissues

**Fig. 3:**
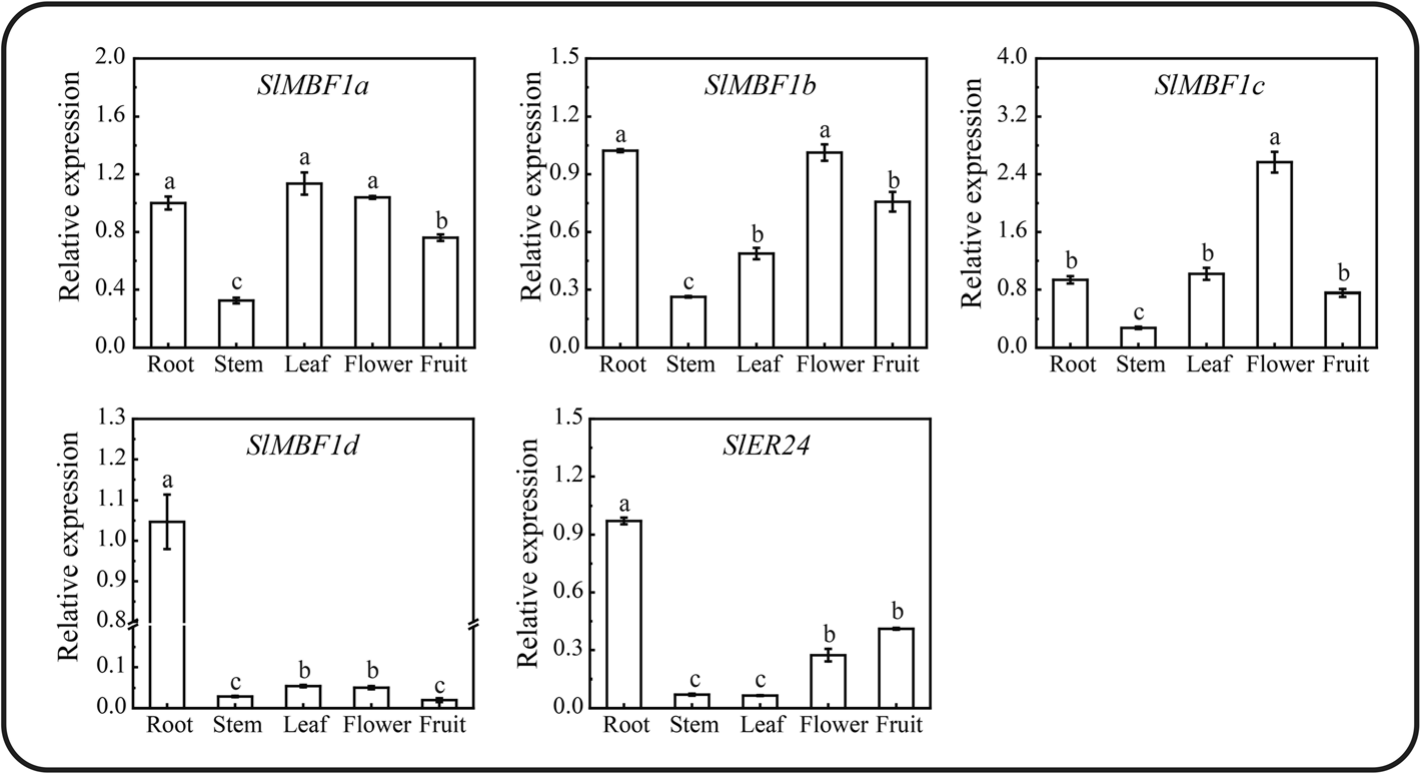
Relative expression analysis of the *SlMBF1* genes in different tissues. The expression levels of *SlMBF1s* in the root, stem, young leaf, flower, and ripe fruit using qRT-PCR analysis. Different letters indicate significant differences (*P* < 0.05)

To understand the potential function of the tomato *SlMBF1* genes, the expression pattern of these five *SlMBF1* genes were examined using qRT-PCR in different tomato organs, including the root, stem, leaf, flower and ripe fruit. As shown in Fig. 3, all of the *SlMBF1* genes were detected in these five tissues. The expression of *SlMBF1a*, *SlMBF1b* and *SlMBF1c* were at relatively high levels in most tissues, but *SlMBF1d* was expressed at relatively lower levels in all tissues. *SlER24* was expressed at relatively lower levels in root, stem and leaves but at relatively high levels in fruit and flower.

### Expression pattern of *SlMBF1* genes under different stress and different plant hormone conditions

**Fig. 4:**
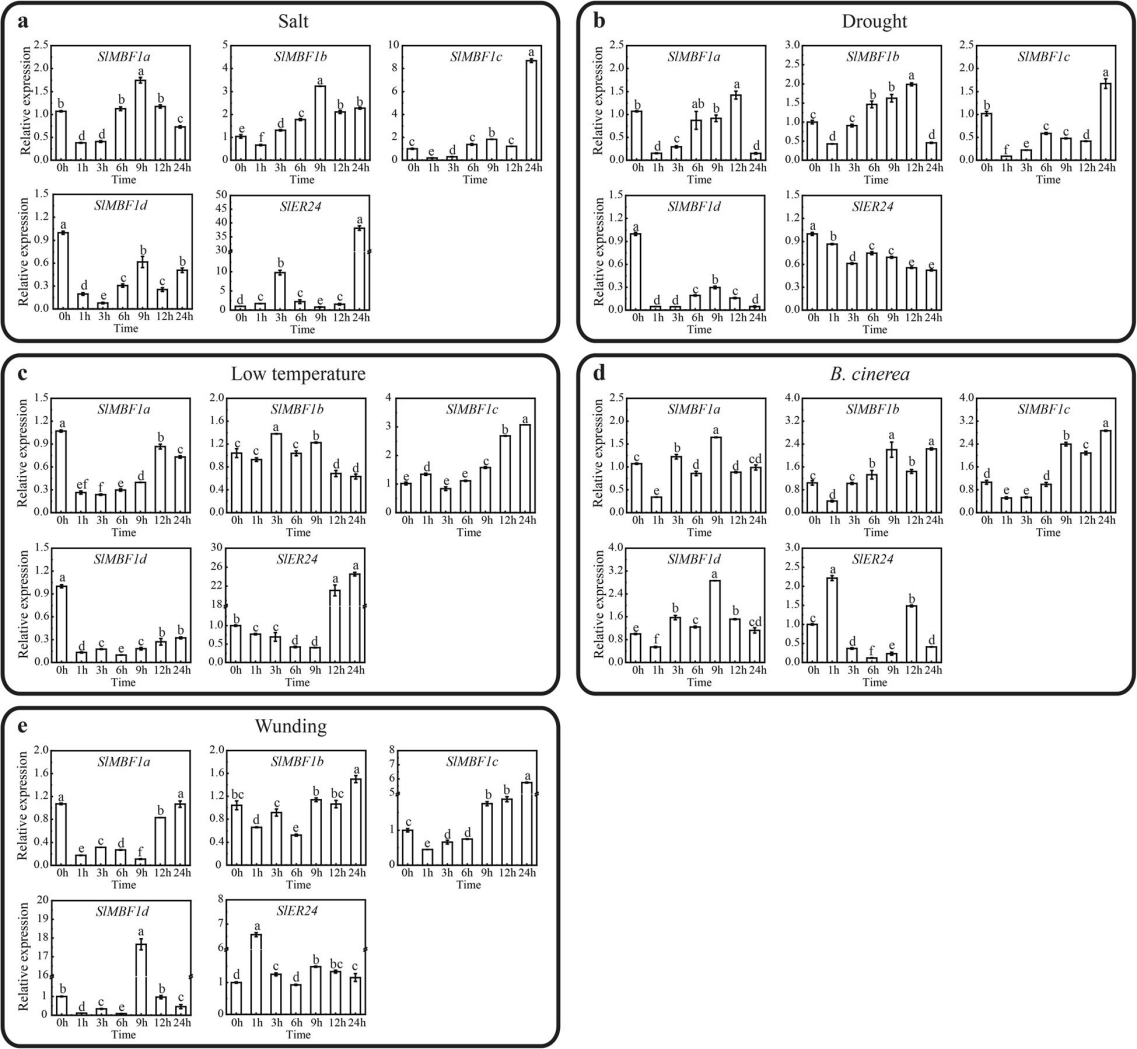
Relative expression analysis of the *SlMBF1* genes under different stress conditions. The expression levels of the *SlMBF1* genes using qRT-PCR analysis under salt, drought, low temperature, *B. cinerea* and wounding stresses. Different letters indicate significant differences (*P* < 0.05)

To explore whether these five *SlMBF1* genes respond to biotic and abiotic stresses in tomato, we examined the expression pattern of the *SlMBF1* genes under different stress conditions, including salt, drought, low temperature, *B. cinerea* and wounding using qRT-PCR (Fig. 4). As expected, most of the *SlMBF1* genes responded to different stress treatments. For example, *SlMBF1c* was induced during the late stage of all stress treatments (Fig. 4). The expression level of *SlER24* was upregulated during the late stage of the salt and low temperature conditions (Fig. 4a, c). The expression level of *SlMBF1a* was initially downregulated then upregulated and then downregulated at the late stage under drought and *B. cinerea* conditions (Fig. 4b, d). Moreover, *SlMBF1b* displayed the same expression trend with *SlMBF1a* under drought conditions (Fig. 4c).

**Fig. 5:**
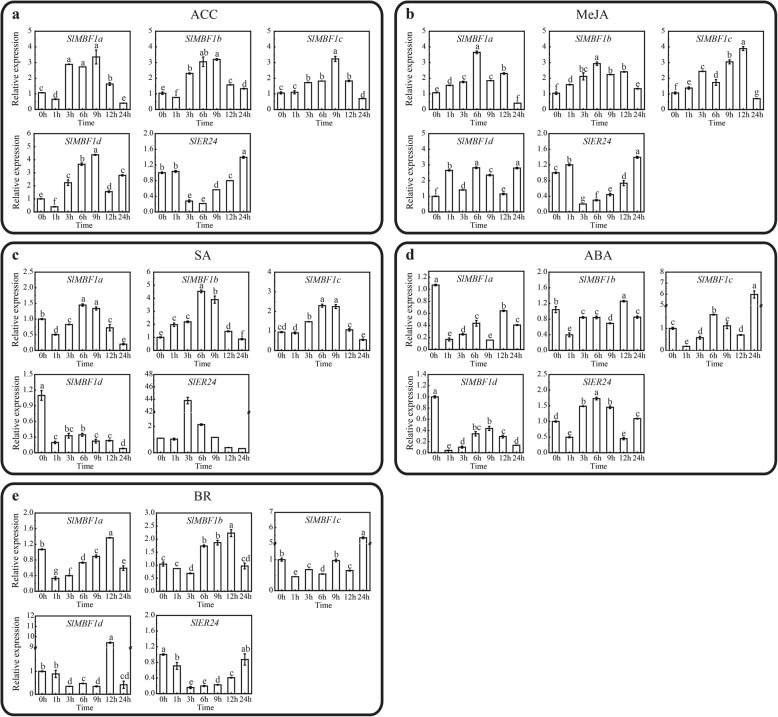
Relative expression analysis of the *SlMBF1* genes under different plant hormone treatments. The expression levels of the *SlMBF1* genes under ACC, MeJA, SA, ABA and BR treatments using qRT-PCR analysis. Different letters indicate significant differences (*P* < 0.05)

To further study how these five *SlMBF1* genes respond to plant hormones in the tomato, we also examined the expression pattern of the *SlMBF1* genes under different hormone treatments, including 1-amino cyclopropane-1-carboxylic acid (ACC), salicylic acid (SA), methyl jasmonate acid (MeJA), abscisic acid (ABA), and brassinosteroids (BR) using qRT-PCR (Fig. 5). As shown in Fig. 5, most of the *SlMBF1* genes responded to different hormones. For example, the expression level of *SlMBF1a* and *SlMBF1c* was initially upregulated then downregulated at late stage under ACC and MeJA conditions. In contrast, the expression level of *SlMBF1a* was initially induced then repressed at the late stage under ACC and MeJA conditions. Some of the *SlMBF1* genes were also induced under the SA, ABA and BR conditions (Fig. 5c, d, e).

### The susceptibility of *SlMBF1c* overexpressing lines to *B. cinerea*

**Fig. 6:**
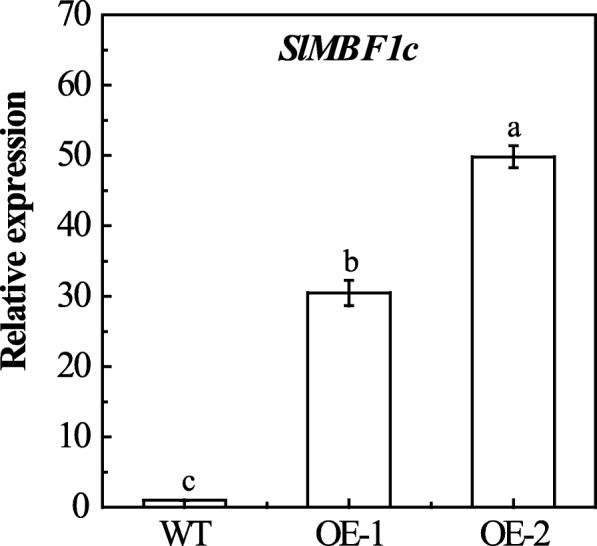
Characterization of the *SlMBF1c* transgenic tomato plants. The leaves of T3 *SlMBF1c*-overexpressing and WT tomato plants were used for the qRT-PCR analysis. The actin gene was used as an internal control to normalize all data. Different letters indicate significant differences (*P* < 0.05)

**Fig. 7:**
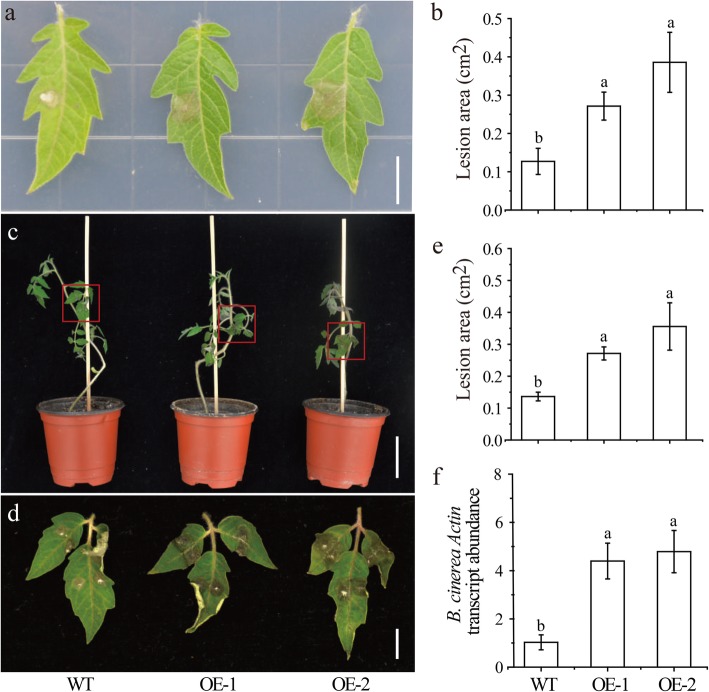
Overexpression of *SlMBF1c* resulted in decreased resistance to *B. cinerea*. **a** The response of wild-type and *SlMBF1c-OE* plant leaves to *B. cinerea* infection at 2 dpi in Petri dishes (Scale bars, 1 cm). **b** The quantification of lesion areas on the leaves shown in (**a)**. **c** and (**d**) The response of whole plants of wild-type and *SlMBF1c-OE* to *B. cinerea* infection at 2 dpi (Scale bars, 5 cm and 1 cm, respectively). **e** The quantification of lesion areas on the leaves shown in (**c**). **f** Relative transcript abundance of the *B. cinerea Actin* in the infected leaves from the whole plant inoculation experiments at 2 dpi. Detached leaves from 5-week-old tomato plants were spotted with 5 μl of spore suspension (10^6^ spores/ml). The results in (**b**), **e** and (**f**) are presented as the mean values ± SD; *n* = six leaves from different plants. Different letters indicate significant differences between treatments (*P* < 0.05)

To investigate the function of *SlMBF1c* in the defense response to *B. cinerea*, we generated *35S::SlMBF1c* transgenic tomato plants (OE) by the Agrobacterium-mediated method. Using kanamycin as selection marker and genomic PCR detection, two independent and homozygous T3 transgenic lines were selected for further assays. These two OE lines display significantly higher expression levels of *SlMBF1c* than the WT plants (Fig. 6). Then, we examined the response of the leaves from 5-week-old OE and WT seedlings to *B. cinerea* infection in Petri dishes, using the method previously described by Du et al., 2017 [17]. As shown in Fig. 7a and b, after infection with *B. cinerea*, the OE leaves showed significantly larger necrotic lesions compared with WT. Moreover, we also conducted the whole plant inoculation experiments. Similarly, the OE plants displayed a sensitive phenotype, compared with WT, after infection with *B. cinerea* (Fig. 7c, d and e). In addition, the expression level of *B. cinerea Actin* was significantly increased in OE plants compared with WT (Fig. 7 f). Taken all together, these results demonstrated that tomato *SlMBF1c* is a negative regulator in the response to *B. cinerea* infection.

### *SlMBF1c* regulates the expression of defense-related genes

**Fig. 8:**
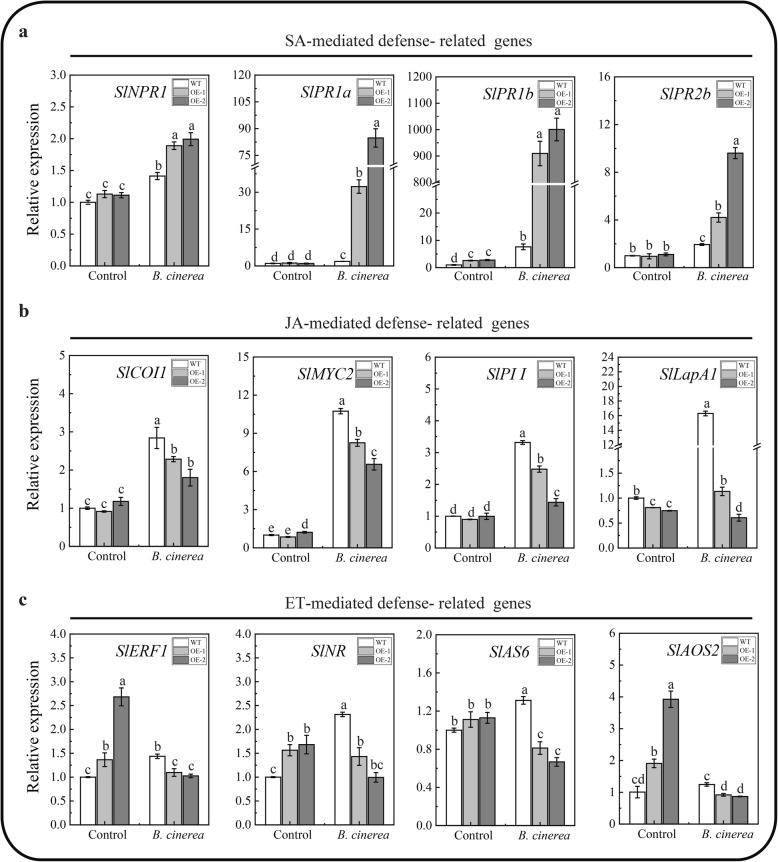
Overexpression of *SlMBF1c* affected the expression of SA-, JA- and ET-mediated signaling genes after *B. cinerea* infection. **a** Expression levels of SA-mediated defense-related genes. **b** Expression levels of JA-mediated defense-related genes. **c** Expression levels of ET-mediated defense-related genes. The inoculation with a spore suspension of *B. cinerea* was done at 10^6^ spores/ml. The sampling time is 1 dpi after infection. Different letters for each defense-related gene indicate significant differences (*P* < 0.05)

To explore the signaling pathways, we analyzed and compared the changes in the relative expression of SA signaling-related genes *Nonexpressed Pathogenesis-Related 1* (*SlNPR1*) and Pathogenesis-Related genes (*SlPR1a*, *SlPR1b* and *SlPR2b*), JA signaling-related genes *Coronatin Insensitive 1* (*SlCOI1*), *Myelocytomatosis Oncogene 2* (*SlMYC2*), *Proteinase Inhibitor I* (*SlPI I*) and *Leucine Aminopeptidase A1* (*SlLapA1*), and ET signaling-related genes *Ethylene Response Factor 1* (*SlERF1*), *Ethylene Receptor* (*SlNR*), *ACC Synthase 6* (*SlACS6*) and *Allene Oxide Synthase 2* (*SlAOS2*) before and after infection with *B. cinerea* using qRT-PCR. As shown in Fig. 8, before infection, the transcript levels of *SlNPR1*, *SlPR1a*, *SlPR2b*, *SlCOI1*, *SlPI I* and *SlACS6* display no significantly difference between the two OE lines and WT. However, the transcript levels of *SlPR1b*, *SlERF1*, *SlNR*, *SlAOS2* were increased slightly and the transcript levels of *SlLapA1* were decreased slightly in the OE lines. After infection with *B. cinerea*, the transcriptional levels of SA signaling-related genes (*SlNPR1*, *SlPR1a*, *SlPR1b* and *SlPR2b*) were elevated significantly in the two OE lines compared with WT (Fig. 8a). However, after infection with *B. cinerea*, the expression levels of the JA signaling-related gene (*SlCOI1*, *SlMYC2*, *SlPII* and *SlLapA1*) and the ET signaling-related genes (*SlERF1*, *SlNR*, *SlACS6* and *SlAOS2*) were significantly decreased in the two OE lines compared with WT (Fig. 8b and c). These results indicated that the overexpression of *SlMBF1c* in the tomato could repress the JA/ET-mediated signaling pathways upon infection with *B. cinerea*.

## Discussion

With the genomes of more species completely sequenced, many regulatory gene families such as the MYB [18], bHLH [19] and WRKY [20] transcription factor families, have been identified. In addition to these transcription factor families, there are also transcriptional co-activator families such as MBF1s. Studies of *MBF1* genes have mainly focused on the regulation of plant growth, development and stress responses in *Arabidopsis* [2, 7, 8]. Although in the year 2007, Sanchez-Ballesta et al. identified four *MBF1* genes in the tomato and analyzed their structures, tissue-specific expression and response to ethylene treatment during fruit development [15], the tomato genome sequence completed in 2012 provides more information for the identification of this gene family [13]. Here, five tomato *MBF1* genes were identified and confirmed based on the completed tomato genome (Fig. 1a). Meanwhile, the more precise and comprehensive bioinformatics analysis (including the chromosomal location, phylogenetic analysis, gene structure, conserved motifs and cis-elements in the promoters) were performed. Notably, we found five exons in the gene structure of *SlMBF1a*, but Sanchez-Ballesta et al. only found four exons. Comprehensive expression levels of these genes in different tissues, responses to different stresses (salt, drought, low temperature, *B. cinerea* and wounding) and different plant hormone conditions (ACC, MeJA, SA, ABA and BR) were also detected (Figs. 4 and 5). More importantly, we identified the biological function of *SlMBF1c* which negatively regulate the tomato resistance to *B. cinerea* (Figs. 6,7 and 8).

In this study, five *MBF1* genes were distributed on five chromosomes of tomato, respectively (Fig. 1a). Compared with three *MBF1s* in *Arabidopsis* [2] and two *MBF1s* in rice, the number of *MBF1s* was greater in the tomato, which means an expansion of *MBF1s* in tomato. A phylogenetic analysis divided these 10 MBF1 proteins into two main branches (Fig. 1b), the same as in previous description [16]. One branch contained subgroup I-A and B, and the other contained subgroup II (Fig. 1b). This result revealed that there are two different evolutionary directions for these MBF1 proteins in tomato, *Arabidopsis* and rice. Importantly, subgroup I-A only includes four MBF1 proteins but did not include any *Arabidopsis* or rice MBF1 proteins (Fig. 1b), which means that this subgroup was lost in *Arabidopsis* and rice and was acquired in tomato after divergence from the last common ancestor. Moreover, the gene structure analysis showed similar intron-exon structures in subgroup I-B and subgroup II but not in subgroup I-A (Fig. 1B), suggesting that the evolutionary dynamics of intron insertion and loss occurred in subgroup I-A of the tomato *MBF1* genes. Previous studies have shown that the yeast *mbf1* mutant was fully/partially rescued by the *MBF1* genes from human, silkworm and *Arabidopsis* [2, 21], which revealed that the functions of the *MBF1* genes are highly conserved. In this study, the motif analysis showed that these MBF1 proteins share similar pattern of motif composition and that all of them have MBF1 and HTH_3 domains (Fig. 1b), which means that the function of MBF1 proteins among tomato, *Arabidopsis* and rice might be similar and conserved.

The expression pattern analysis in different tissues, stresses and plant hormones is usually used to predict the potential functions of genes in plant growth, development and the responses to stresses. Through the expression pattern analysis, we found that all of the *SlMBF1s* genes were expressed in the five tissues, and most of them had much higher expression in the flower and leaf (Fig. 3). In addition, GA response element was found in the promoter regions of these five *SlMBF1* genes and IAA response element also in the promoter regions of *SlMBF1a*, *SlMBF1b* and *SlER24* (Fig. 2). These results indicated that *SlMBF1s* might be involved in plant growth and development. Besides the roles in plant growth and development [14], *MBF1* genes also participate in the responses to abiotic and biotic stresses, such as salt, drought, temperature and pathogens [5–12]. Indeed, several stress-related elements (drought, low temperature, ABA, defense and stress, JA and SA response elements) were found in the promoter regions of these *SlMBF1s* (Fig. 2). In addition, *SlMBF1* genes were induced by abiotic and biotic stresses (e.g. salt, drought, cold and *B. cinerea*) and by stress-related hormones (e.g. ABA, SA, JA and ACC) (Figs. 4 and 5). These results indicated that these *SlMBF1* genes were involved in the responses to stresses with the functions similar to the *MBF1s* from other species [7–12].

The tomato is an important economic and vegetable crop. However, *B. cinerea* seriously limits the yield of tomato [22]. In this study, the expression of *SlMBF1c* was significantly induced by *B. cinerea*, wounding and defense-signaling related hormones (Figs. 4 and 5). Additionally, several defense related elements were also found in the promoter of *SlMBF1c* (Fig. 2). Moreover, overexpressing the *AtMBF1a* gene in *Arabidopsis* confers increased resistance under the infection by *B. cinerea* [7]. In order to clarify the function of *SlMBF1c* in the defense response, tomato plants overexpressing *SlMBF1c* were generated. To our surprise, the transgenic lines displayed a sensitive phenotype, as compared with WT, under infection with *B. cinerea* (Fig. 6). The finding that *SlMBF1c* regulates the resistance to *B. cinerea* is distinct from the function of its *Arabidopsis* homolog. This phenomenon might be due to the evolutionary differences between *SlMBF1c* and *AtMBF1a*, because *SlMBF1c* belonged to subgroup I-A, but *AtMBF1a* to subgroup I-B in the phylogenetic tree (Fig. 1b).

Previous studies showed that *B. cinerea* can activate the SA signaling pathway to promote its pathogenicity in plants [23, 24]. Meanwhile, plants can activate the JA/ET-mediated defense responses against *B. cinerea* infection [23, 25, 26]. However, the SA signaling pathway can antagonize the JA/ET signaling pathways in plants under *B. cinerea* infection [23, 25]. In our study, under control condition, only *SlPR1b* in the SA signaling pathway showed slightly higher expression in the OE lines compared with WT, but under *B. cinerea* infection, all of *SlNPR1*, *SlPR1a*, *SlPR1b* and *SlPR2b* were significantly up-regulated in OE lines (Fig. 8a). Moreover, the overexpression of *SlMBF1c* further promote the expression levels of the SA signaling pathway genes, especially *SlPR1a* and *b* (Fig. 8a). On the contrary, under control condition, only *SlLapA1* in the JA signaling pathway showed slightly lower expression in the OE lines compared with WT; but under *B. cinerea* infection, all of *SlCOI1*, *SlMYC2*, *SlPI I* and *SlLapA1* were significantly down-regulated in OE lines (Fig. 8b). These results suggested that the JA-mediated defense responses in infected OE lines was seriously suppressed by the highly activated the SA signaling pathway under *B. cinerea* infection (Fig. 8b) [26]. In addition, although under control condition, the ET-mediated defense genes (*SlERF1*, *SlNR*, *SlACS6* and *SlAOS2*) showed higher expression in the OE lines compared with WT (Fig. 8c), all of these genes were significantly down-regulated in the infected OE lines, indicating that the ET signaling pathway in the infected OE lines was also greatly suppressed by the highly activated SA signaling (Fig. 8c). Taken together, these results clarified that the *SlMBF1c*-overexpressing tomato plants displayed a sensitive phenotype due to the strongly activated SA pathway which antagonized the JA/ET-mediated defense responses under *B. cinerea* infection.

*NPR1* is not only a master regulator of SA signaling, but also a key regulator in the antagonism between SA and JA through suppressing the JA signaling gene *PI I* [23, 27]. Indeed, the expression level of *SlNPR1* was increased and *SlPI I* repressed in the infected OE lines (Fig. 8a-b), suggesting that *SlMBF1c* could activate *SlNPR1* to repress *SlPI I* in infected OE lines. This result is consistent with the previous study [26]. However, in our study, the expression levels of *SlPR1a* and *SlPR1b* were more dramatically increased than *SlNPR1* in the infected OE lines, and *SlLapA1* more dramatically decreased than *SlPI I* (Fig. 8a-b). Therefore, it will be interesting to clarify that how *SlPR1a* and *SlPR1b* are induced and whether *SlPR1a* and/or *SlPR1b* are the new key regulators to suppress *SlLapA1* in the antagonism between SA and JA signaling pathways when *SlMBF1c* is being overexpressed in the tomato under *B. cinerea* stress condition in the future studies.

## Conclusion

In this study, five *SlMBF1* genes including *SlMBF1d* newly-found were identified and confirmed in the tomato genome. The analysis of phylogenetic tree, gene structures and protein motifs revealed that MBF1 proteins are conserved among tomato, *Arabidopsis* and rice and expanded in the tomato. The cis-elements in the promotors, tissue specific expression pattern and responses to stresses and hormones suggested that the *SlMBF1s* might participate in plant growth and development and stress responses in the tomato. Finally, transgenic experiments showed that *SlMBF1c* negatively regulate the tomato resistance to *B. cinerea* through enhancing SA-signaling genes and repressing the genes in the JA/ET-mediated pathways.

## Methods

### Identification of *MBF1* genes in the tomato

To identify the *SlMBF1* gene family members from the entire tomato genome, three AtMBF1 proteins were used as query sequences for Blastp searches with an e-value of 10^− 10^ against the predicted tomato proteins. In addition, the Hidden Markov Model (HMM) profile of MBF1 (PF08523.9) and HTH_3 (PF01381.21) from the Pfam database (http://pfam.janelia.org) were also applied as queries to search the *MBF1* genes from the tomato genome database (http://solgenomics.net; ITAG Release 3.20). In order to identify the conserved domains, five candidate genes were further confirmed due to the presence of both the MBF1 (PF08523.9) and HTH_3 (PF01381.21) domains using the Pfam database and SMART database (http://smart.embl-heidelberg.de).

The MBF1 proteins in the representative model plants *Arabidopsis* and rice were downloaded from The *Arabidopsis* Information Resource database (https://www.arabidopsis.org) and the Rice Genome Annotation Project Database (http://rice.plantbiology.msu.edu/).

### Phylogenetic analysis

The multiple sequence alignment was constructed by Clustal W (version 1.81, a resident software, European Molecular Biology Laboratory, Heidelberg, Germany.) with default parameters [28]. Full sequences of five SlMBF1, thress AtMBF1 and two OsMBF1 proteins were used to construct the phylogenetic tree using MEGA v7.0 [29]. The Neighbor-Joining method was used with the following parameters: Poisson correction, pairwise deletion, and bootstrap (1000 replicates; random seed) [30].

### Analysis of physical properties, chromosomal localization, gene structure, conserved motif recognition and response elements in promoter regions

Physical properties such as theoretical protein isoelectric point (pI) and molecular weight of the SlMBF1 proteins were calculated using the ExPASy server’s Compute pI/Mw tool (http://web.expasy.org/compute_pi/) [31]. The information of the chromosomal locations and gene structures were downloaded from the tomato genome database. The conserved motifs were analyzed using MEME database (http://meme-suite.org/) [32]. Additionally, the response elements in the promoter regions were analyzed using the PlantCARE database (http://bioinformatics.psb.ugent.be/webtools/plantcare/html/) [33]. The chromosomal locations were visualized by Mapchart 2.30 software [34]. The gene structures, conserved motifs and response elements in the promoter regions were visualized by GSDS Server 2.0 (http://gsds.cbi.pku.edu.cn/).

### Plant materials and growth conditions

Tomato (*Solanum lycopersicum* L. cv ‘SN1’ [35]) seedlings were grown in a biotron at Shandong Agricultural University with a 16 h light (28 °C)/8 h dark (22 °C) photoperiod (18.5 μmol m^− 2^ s^− 1^). Four-week-old tomato seedlings were used for all types of treatments.

### Different stresses and hormone treatments

For the salt and drought stress treatment assays, tomato plants (4-week-old) were transferred into the 10 L tanks containing half-strength Hoagland nutrient solution and were maintained in this system for one week before supplementation with NaCl (150 mM) and Polyethylene glycol 8000 (20%), as previously described [36]. The tomato plants were transferred to the incubator for cold treatment at 4 °C. The seedling leaves were pressed with hemostatic forceps for the wounding treatment. The inoculation of the tomato plants with *B. cinerea* (B05.10) was performed as previously described [17, 23, 37], with minor modifications. The seedling leaves were spotted with a single 5-μl droplet of *B. cinerea* spore suspension (10^6^ spores/ml) for the pathogen treatment. For the hormone treatments, the seedling leaves were sprayed with ACC (100 μM), SA (2 mM), MeJA (100 μM), ABA (100 μM) and BR (200 μM). The leaves from different tomato plants were collected for the qRT-PCR analysis.

### RNA isolation and quantitative real-time PCR analysis

The total RNA from tomato leaves was extracted with TRIzol Reagent (Invitrogen, Carlsbad, CA, USA) according to the manufacturer’s instructions. The first-strand cDNA was synthesized from one microgram of total RNA using a reverse transcriptase system (Thermo, Beijing, China), according to the manufacturer’s instructions. The reactions were performed using the SYBR Mixture (Juheme) with an Applied Biosystems 7500 real-time PCR system (Applied Biosystems). The PCR assays were conducted with the following parameters: 95 °C for 30 s; 40 cycles of 95 °C for 30 s, 60 °C for 15 s, and 72 °C for 15 s. All of the primers that were used in the qRT-PCR analysis are listed in Additional file 4: Table S4, some of which came from the previous studies [24, 38–40]. The tomato *Actin2* gene was used as the internal control. The results were calculated using the 2^−ΔΔCt^ method [41]. All of the qRT-PCR assays were conducted in three biological replicates and each biological replicate had three technical replicates.

### Vector construction and plant transformation

For the construction of the overexpressing *SlMBF1c* vector, the entire *SlMBF1c* coding sequence was amplified using the primers SlMBF1c-F: TATCACAAGACTGGGAGC and SlMBF1c-R: GTCGTACTACTAGAGGCA. Then, the amplified products were digested with *XbaI* and *KpnI* sites and inserted into the pBI121 vector under the control of the 35S promoter. The *35S: SlMBF1c* construct was transferred into the Agrobacterium strain LBA4404 by electroporation, and the Agrobacterium-mediated tomato transformation was performed following the protocols described by Fillatti et al. [42].

### Statistical analysis

All of the error bars for expression levels, represent the standard deviation (SD) which came from three technical replicates, except that in the phenotypic analysis of OE lines which came from six biological replicates. The analysis of significance level was performed with the Student’s *t*-test at *p <* 0.05 using Excel 2010 (Microsoft Cooperation, Washington, NJ, USA).

## Supplementary information

**Additional file 1: Table S1.** Molecular properties of *SlMBF1* gene family in tomato.

**Additional file 2: Table S2.** Ten conserved motifs sequences and the bit score means information content from all MBF1 proteins from tomato, *Arabidopsis* and rice.

**Additional file 3: Table S3.** The conserved domains information of five tomato MBF1 protein.

**Additional file 4: Table S4.** Primers used for qRT-PCR.

## Data Availability

The data that support the results are included within the article and its additional file. Other relevant materials are available from the corresponding authors on reasonable request.

## Abbreviations

aa: amino acid
ABA: Abscisic acid
ACC: 1-Amino cyclopropane-1-carboxylic acid
AtMBF1: *Arabidopsis thaliana* Multiprotein bridging factor 1
*B. cinerea*: *Botrytis cinere*a
BR: Brassinosteroids
Da: Dalton
JA: Jasmonic acid
MBF1: Multiprotein bridging factor 1
MEGA: Molecular Evolutionary Genetics Analysis
MeJA: Methyl jasmonate
NJ: Neighbor-Joining
OE: Overexpression
OsMBF1: *Oryza sativa* Multiprotein bridging factor 1
pI: protein isoelectric point
SA: Salicylic acid
SlMBF1: *Solanum lycopersicum* Multiprotein bridging factor 1
